# ClustVis: a web tool for visualizing clustering of multivariate data using Principal Component Analysis and heatmap

**DOI:** 10.1093/nar/gkv468

**Published:** 2015-05-12

**Authors:** Tauno Metsalu, Jaak Vilo

**Affiliations:** Institute of Computer Science, University of Tartu, J. Liivi 2, 50409, Tartu, Estonia

## Abstract

The Principal Component Analysis (PCA) is a widely used method of reducing the dimensionality of high-dimensional data, often followed by visualizing two of the components on the scatterplot. Although widely used, the method is lacking an easy-to-use web interface that scientists with little programming skills could use to make plots of their own data. The same applies to creating heatmaps: it is possible to add conditional formatting for *Excel* cells to show colored heatmaps, but for more advanced features such as clustering and experimental annotations, more sophisticated analysis tools have to be used. We present a web tool called ClustVis that aims to have an intuitive user interface. Users can upload data from a simple delimited text file that can be created in a spreadsheet program. It is possible to modify data processing methods and the final appearance of the PCA and heatmap plots by using drop-down menus, text boxes, sliders etc. Appropriate defaults are given to reduce the time needed by the user to specify input parameters. As an output, users can download PCA plot and heatmap in one of the preferred file formats. This web server is freely available at http://biit.cs.ut.ee/clustvis/.

## INTRODUCTION

With the advent of high-throughput experiments, scientists are often confronted with multivariate data, usually presented as a matrix. This type of data can come from a variety of sources, for example gene expression studies where looking at specific genetic pathways is of great interest (1). In addition to that, image analysis algorithms can be used on high resolution images to extract features relevant for clinical cancer prognosis (2). Heatmap and Principal Component Analysis (PCA) are the two popular methods for analyzing this type of data.

Heatmap is a data matrix visualizing values in the cells by the use of a color gradient. This gives a good overview of the largest and smallest values in the matrix. Rows and/or columns of the matrix are often clustered so that users can interpret sets of rows or columns rather than individual ones. PCA is a method where a multivariate data set is linearly transformed into a set of uncorrelated variables, ordered in descending manner by the variance explained (3). This way, one can interpret first few components that often explain large amount of the variation.

The observations under investigation often have pre-defined experimental annotation groups and adding this information to both of the plots would make the interpretation easier. For example, it would be possible to see whether some of the sample groups overlap with each other or form separate clusters. Without specific statistical software, this type of analysis is usually not straightforward and needs the software to be installed and configured.

For example, conditional formatting can be applied in *MS Excel* to show a color gradient, but it is not easy to cluster or annotate the matrix. A proprietary *Excel* add-in called *NumXL* allows the calculation of principal components, but showing the groups is again more complicated. The similar applies to Galaxy web server (galaxyproject.org) and popular statistics software (for example R, SAS, Statistica) which allow performing PCA and plotting heatmap but showing annotations and customizing the output is still quite complicated for an average user. An Excel embedded application called imDEV (4) also allows to make PCA plots and heatmaps but needs installation on a specific platform. None of the alternative tools described allows to load a public gene expression data set directly, there is always some pre-processing needed outside the tool.

We present a web tool called ClustVis that aims to make this type of analysis easier. The user can upload his/her own data or alternatively use one of the built-in public gene expression microarray data sets from ArrayExpress (5). Both heatmap and PCA plot can be generated and modified in a variety of ways using an intuitive user interface.

## MATERIALS AND METHODS

### Implementation

ClustVis is written using Shiny web application framework (R package version 0.10.2.1) for R statistics software (6). We have used BoxPlotR (7) as an example application. ClustVis uses several R packages internally, including ggplot2 (8) for PCA plot, pheatmap (R package version 0.7.7) for plotting heatmap, pcaMethods (9) for different methods to calculate principal components using data that contain missing values, FactoMineR (10) to calculate confidence ellipses, RColorBrewer (R package version 1.0–5) (11) for color palettes, shinyBS (R package version 0.20) for tooltips, g:Profiler (12) for converting gene names etc. ClustVis is freely available without login requirement at http://biit.cs.ut.ee/clustvis/.

### Detecting delimiter and number of annotations

For user-uploaded data sets, ClustVis detects automatically both, the delimiter and the number of annotation rows from the data by default. In order to find the delimiter, it counts for each possible delimiter (comma, tabulator, semicolon) how many times it appears on each row. We use the heuristic where minimum is taken over all rows and the delimiter with the greatest score is chosen as the right one.

When finding number of annotations, at first, all rows containing any non-numeric data are considered annotations. If the last row before non-integer numeric data contains only integers (for example, sex coded as 0 for male and 1 for female), this one, as well as all preceding rows are considered annotation rows.

### Principal components

Principal components are calculated using one of the methods in pcaMethods (9) R package. The default method is SVD with imputation which performs imputation and Singular Value Decomposition (SVD) iteratively until estimates of missing values converge. Nipals PCA will iteratively find components by leaving out missing values when calculating inner products. Probabilistic PCA is also an iterative method that uses density model for calculating principal components.

### Data pre-processing

Row scaling uses one of the methods from pcaMethods (9) R package. Unit variance scaling method divides the values by standard deviation so that each row has variance equal to one. Pareto scaling method divides the values by the square root of standard deviation. Vector scaling divides the values by the Euclidean norm of the values (square root of sum of squared values).

### Heatmap

Heatmap is plotted using pheatmap R package (version 0.7.7). The source code of pheatmap package was slightly modified to improve the layout and to add some features. The package uses popular clustering distances and methods (13) implemented in *dist* and *hclust* functions in R. The list of distances include correlation (defined additionally as correlation subtracted from 1), Euclidean, maximum, Manhattan, Canberra and binary distance. Linkage methods include single, complete, average, McQuitty, median, centroid and Ward linkage. When k-means clustering has been selected, the R function *kmeans* is used.

### Public data sets and pathways

ClustVis includes multiple popular public data sets for testing purposes: NKI breast cancer data set (14,15), Wisconsin diagnostic breast cancer data set (16) and Fisher's Iris data set (17).

In addition to small data sets, we used the last version of Multi Experiment Matrix—MEM (18). MEM contains a very large collection of public gene expression matrices from ArrayExpress (5), together with annotation tracks where available. Genetic pathways were downloaded from g:Profiler web tool (12). From Gene Ontology, only biological processes were included. Microarray platforms and genetic pathways cover currently 17 species.

## RESULTS

### Overview of the user interface

ClustVis is aiming for an intuitive user interface. The tool can be accessed by any modern browser (Google Chrome, Mozilla Firefox, Internet Explorer, Safari). The user can click on different tabs to move between the steps of analysis. Results from visited tabs are automatically cached for fast retrieval. Parameters are inserted by using widgets such as drop-down menus, text boxes, sliders etc. On each step, reasonable default settings are given whenever it is appropriate to save user's time. When changing some of the settings, results are re-calculated and shown automatically.

### Data input

#### Filtering and pre-processing

Both, PCA as well as heatmap, take a numeric data matrix as input where multiple dimensions (e.g. genes) are measured in multiple observations (e.g. samples). Often, observations also have *a priori* grouping defined by annotations that is useful to be shown on the plot. For example, one may be interested in comparing diseased and normal samples marked differently. In this case, one binary annotation track will be available.

To make data input easier for the end user, we have defined the input file format that includes both, annotations as well as numeric data, in a single file. Such file can be easily manually created in a spreadsheet program (e.g. *MS Excel*). Observation names are listed in the first row, followed by any number of annotations and a numeric matrix. The first column is reserved for annotation labels and dimension labels.

There are two ways of uploading own data: by uploading a delimited file or copying and pasting the contents of the file to ClustVis text box. In both cases, the delimiter and the border between annotations and numeric data are detected automatically from the data. In the rare cases when it does not work correctly, it is possible to specify them manually. Instead of loading own data, it is also easy to load a sample data set or choose a public gene expression microarray data set. In order to search for more relevant public data sets from the list, it is possible to set the minimum number of annotation tracks. Data sets that have no annotations are excluded by default.

Hybrid select/textbox is used to find a gene expression data set, so that a user can make queries using one or more keywords of interest and then choose from the list that contains them. It is possible to use data set title, ID and/or platform ID for querying data sets from one of the 17 species.

In order to limit the number of rows shown on heatmap, the first option is to select one functional category to be included. The list contains KEGG (19) and Reactome (20) pathways and GO (21) biological processes that can be browsed using similar hybrid select/textbox as used for data sets. Both, the names and IDs of the categories are provided.

Another option to limit the number of rows is to cluster the genes using k-means first (13). A user can specify the number of clusters; cluster centers are shown on the heatmap. If the cluster of interest has been detected, the user can explore it further by specifying the cluster ID.

Public data sets often contain annotation tracks that are not informative. For example, some property can be common for all samples in the data set and, therefore, appear as constant. Some of the others can have different value for each sample and as such are not defining any interesting groups. Therefore, annotation tracks that have at least two and not more than eight levels are shown by default. This can be changed by a user. In order to make it easier for users to set these thresholds, number of levels in each annotation track of the original data set is also shown.

Sometimes, users may want to analyze subsets of observations without the need to filter them manually and upload the subset as a new data set. Some larger public data sets may also contain subsets of interest. ClustVis has filtering option for these cases: the user can select one or more annotation tracks and levels in them to apply filtering. Filtered table is shown to the user so that he/she can correct it immediately if anything has gone wrong.

Next, it is possible to aggregate observations belonging to the same annotation group by taking median or mean. In this case, each group will be represented with just a single point, making the plot visually better in some situations. On the other hand, it is not possible to estimate the variability inside the groups this way.

Dimensions can be optionally centered and scaled before doing PCA and heatmap. This is highly recommended if they have different units, otherwise dimensions with larger units would get unreasonably higher impact and can give misleading results.

Many real-world data sets are lacking some numeric values due to technical issues encountered during data collection. Originally, PCA requires the whole input matrix to be filled and clustering of heatmap works also better for complete matrices. Therefore, missing values need special treatment. We use pcaMethods R package (9) that includes methods to impute missing data or calculate principal components directly from incomplete data set. The latter way can also be reversed to predict a complete data set which can be used for heatmap.

### PCA output

PCA plot shows a scatterplot with axes corresponding to the two different principal components. These do not have to be the first two showing the largest variance. Sometimes, first components are related with technical variation such as batch effect (22). In this case, the user can look at other components that can be attributed to more informative sources of variability.

By definition, direction of each single principal component is not uniquely determined. In other words, one can mirror all points along one of the axes without changing the meaning of the plot. In ClustVis, the direction is determined so that median of each component is non-negative. A user can change the direction using a checkbox, possibly to make the plot easier to compare with PCA plot created with the help of some other tool.

**Figure 1.**
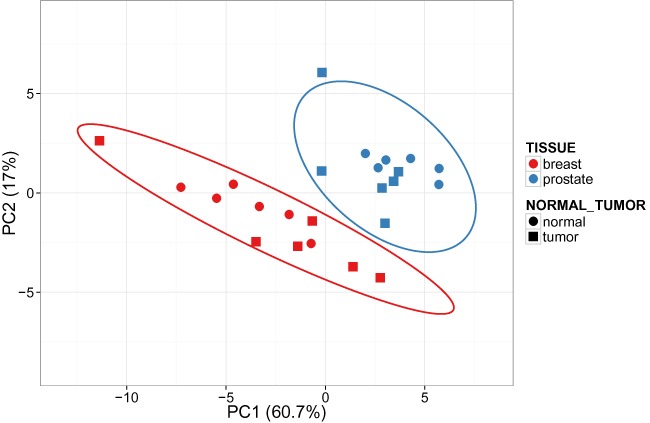
PCA plot of stromal molecular signatures of breast and prostate cancer samples. Ellipses and shapes show clustering of the samples.

The possibility to mark annotation groups with different colors and/or shapes is an important feature. This makes comparing groups with each other possible. The tool offers an option to choose multiple annotation tracks to combine them. Prediction ellipses can be drawn in order to get a better idea how much groups differ from each other (see Figure 1). The confidence level that determines the size of ellipses can also be changed. For example, a confidence level of 0.95 defines the ellipses in the manner that approximately 95% of the new observations from that group would fall inside the ellipse.

There are multiple options available for changing the final layout and appearance of the plot. A user can change axes’ labels, font size, plot width and ratio, point size, color and shape, ellipse line width and type, as well as the legend position. Optionally, one can also show sample IDs on plot to find specific points and hide percentage of variance explained.

### Heatmap output

**Figure 2.**
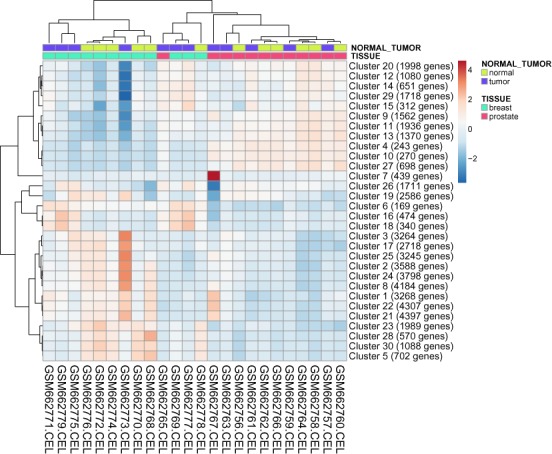
Heatmap of stromal molecular signatures of breast and prostate cancer samples. Annotations on top of the heatmap show clustering of the samples.

Heatmap shows a data matrix where coloring gives an overview of the numeric differences. In ClustVis, hierarchical clustering can be optionally applied to dimensions and/or observations. Users can choose which clustering method to use (if any). Linkage method is another parameter that affects the results and can be changed. Ordering of the clustering tree can be configured and annotation tracks can be placed at the top of the matrix to interpret them in conjunction with the clustering tree (see Figure 2).

A good color scheme is an essential factor for correct interpretation of the heatmap. The user can choose between a variety of diverging and sequential color schemes (11). Diverging palettes fix colors in both lower and higher end of the data and in the middle; they are better suited for data that range to both, negative as well as positive, directions (e.g. difference from some reference). Sequential palettes fix the lowest and the highest value; they are more appropriate for non-negative data (e.g. percentage between 0 and 100). There is also an option to reverse the scheme colors and set the limits of color range manually.

Other options are related to the changes in the layout and appearance, such as the font size of observations and dimensions (if shown); font size and precision of numbers in the cells (if shown); cell border color; plot width and ratio and whether annotation titles are shown.

### Export of analysis results

Both PCA plot and heatmap can be downloaded using one of the three formats (PDF, EPS or SVG). It is also possible to download the initial data in case a public data set was used that was not available to the user before. Intermediate results, including processed data, PCA scores, PCA loadings and variance explained by the PCA components can be downloaded as well.

Both PCA and heatmap show an example caption above the plot that describes the data processing and visualization using full sentences. This makes it more convenient to describe the plot in an article written by a user of ClustVis.

Due to the large number of settings available there is also an option to save them and generate a link that loads pre-saved settings. Subsequently, the link can be sent to collaborators to show current findings or keep it to return to the same data set later.

## DISCUSSION AND FUTURE DEVELOPMENTS

Both PCA and heatmap can be used to estimate whether pre-defined groups form separate or overlapping clusters. For example, let us look at a data set about stromal molecular signatures of breast and prostate cancer samples (23). 54675 genes are measured, we aggregate them into 30 clusters using k-means clustering. From the PCA plot, we can see that breast and prostate cancer samples form separate clusters (see Figure 1). Inside both groups, we cannot differentiate between normal and tumor samples, but we can see that normal samples vary less than tumor samples. Similar conclusions, except about the variation, can be drawn using annotations above the heatmap (see Figure 2). From the heatmap, we can find two samples (GSM662767 and GSM662773) that look different from other samples and are worth further investigation.

When applying software packages to visualize PCA on scatterplot, default options are often used so that *x*- and *y*-axis have different scale. This is generally not a good practice if the scales of first components are very different because the plot can be intuitively used for approximating Euclidean distances between points. Using different scales, units of one of the components are magnified in relation to the other and it is very hard to compare distances. In ClustVis, *x*- and *y*-axis are always forced to have the same scale.

When interpreting the clustering of heatmap, it is important to pay attention to which objects are merged into the clustering tree first and not to the exact order of rows and/or columns. Any two branches can be swapped without changing the meaning of the tree. A user can change branch re-ordering method to make the heatmap visually more attractive.

There is an idea to make the tool more interactive. For example, we could have 3D PCA plot so that the user can see three first principal components instead of two and rotate the points. We could also add links or tooltips for each point or heatmap cell which will contain information on each specific observation. Although these ideas look promising and are worth considering in the next version of ClustVis, we have concentrated first on enabling users to easily generate publication-quality plots, rather than providing fully dynamic interactive exploratory environment.

Currently ClustVis limits the input file size and does not allow matrices of very high dimensionality to be visualized on a heatmap. This is mainly due to the performance issues: huge matrices take longer to upload and calculate clustering and, therefore, make the tool slower and inconvenient to use. In addition to that huge heatmaps are usually not so easily readable. These limits may be reconsidered in the next versions of ClustVis.
